# Who should finance science? A consideration about publication costs

**DOI:** 10.5935/0004-2749.2024-1010

**Published:** 2024-06-20

**Authors:** Newton Kara-Junior

**Affiliations:** 1 Department of Ophthalmology, Universidade de São Paulo, São Paulo, SP, Brazil

Big research groups do not need to worry about the costs of publishing their studies, as they generally have sufficient public or private funding to cover execution and publication expenses. Thus, journals with a very high-impact factor (IF) can charge authors any publication fees they want, and the cost will not burden the researcher.

In Brazil, most researchers linked to universities face the challenge of integrating research, teaching, and extension activities. In addition, they may receive grants from public funding agencies to cover some of the costs of conducting their studies. Conversely, *stricto sensu* postgraduate students often receive public research grants to have the financial conditions to dedicate themselves to research.

However, the students end up using their grant money to cover the publication fees for their studies. As international publishers manage many scientific journals, public money goes to them. Thus, we consider the government and researchers to be the primary financiers of science in Brazil. Those who benefit from scientific research are the large publishing companies that can charge authors exorbitant fees.

When financing science, the government’s objective is to stimulate high-quality research and innovation to improve the country’s technological performance. Along with research grants, it relies on *Coordenação de Aperfeiçoamento de Pessoal de Nível Superior* (CAPES) (a government body that evaluates postgraduate programs), directing postgraduate programs to value publications in journals with good IF and innovations/patents. CAPES has consistently met the country’s needs by providing subsidies for publications in national journals and sponsoring those that do not charge authors.

Therefore, the government supports national researchers and journals. However, we believe it could help more and consider it a waste for young researchers to use their research grants to pay publication fees. When evaluating the postgraduate program, CAPES itself requires publications in high-impact factor journals, which generally charge high publication fees^(1^, ^2)^.

We suggest that CAPES uses its “bargaining” power to negotiate discounted publication rates directly with journals and publishers. This project would provide a list of recommended journals with strong editorial practices and partners. A financing option would be to distribute vouchers to postgraduate programs to pay publication fees at a discount previously negotiated with journals accepting the proposal.

The list of journals recommended by CAPES could also include journals that do not charge authors or readers (called diamond). A possible addition would be a bonus in the evaluation of the postgraduate program for publications in journals in this category. Furthermore, this recommendation list would protect authors from predatory journals.

Thus, by directing funding more efficiently, the government would further support researchers, especially those who do not work exclusively on scientific production.
